# Quantifying the burden of Hypercholesterolemia among Adolescents in India

**DOI:** 10.1093/eurpub/ckac130.040

**Published:** 2022-10-25

**Authors:** K Kirti, SK Singh

**Affiliations:** Department of Biostatitics and Demography, International Institute for Population Sciences, Mumbai, India

## Abstract

Hypercholesterolemia is a kind of hyperlipidemia in which an individual's blood contains excessive non-high-density lipoprotein (non-HDL) cholesterol and low-density lipoprotein (LDL) cholesterol, which has emerged as a significant covariate of coronary heart disease. Descriptive, bivariate and multivariable regression analyses were used to unearth the current hypercholesterolemia levels, probable risk factors, and its impact on other metabolic diseases among adolescents using data on 35,830 adolescents aged 10-19 years from the Comprehensive National Nutrition Survey, India, 2016-18. Findings suggest that the mean lipid levels for total cholesterol, LDL, HDL, and triglycerides were 140.6, 84.1, 47.3, and 95.3, respectively, with females bearing the higher burden. The study further identified early adolescents, urban residents, and overweight individuals at a higher risk of having elevated non-HDL. Western and Eastern regions bore higher LDL levels. Further, for a unit increase in TSFT risk of having high LDL increased by 2.55 times. Zinc deficits are at 2.13 times higher risk compared to zinc sufficient. Adolescents consuming unhealthy diets were at higher risk of elevated LDL. The study contends that it is essential to prevent the increasing levels of lipid profiles among Indian adolescents. Vitamin and mineral deficiencies and unhealthy dietary habits are significantly associated with high LDL and non-HDL levels. In the longer run, this might cause the early onset of complex cardiometabolic disorders, which would disrupt the individual's social and economic well-being. Hence, appropriate interventions are needed to curtail these early onsets by primarily focusing on adolescents.

**Key messages:**

- Present study is primarily focussed upon the cholesterol levels among adolescents in India.
- How this imbalance in cholesterol levels among adolescents would affect the chronic conditions among adolescents in India.

